# Targeting the CD27-CD70 Pathway to Improve Outcomes in Both Checkpoint Immunotherapy and Allogeneic Hematopoietic Cell Transplantation

**DOI:** 10.3389/fimmu.2021.715909

**Published:** 2021-09-22

**Authors:** Forat Lutfi, Long Wu, Sarah Sunshine, Xuefang Cao

**Affiliations:** ^1^ Marlene and Stewart Greenebaum Comprehensive Cancer Center, University of Maryland Medical Center, Baltimore, MD, United States; ^2^ Marlene and Stewart Greenebaum Comprehensive Cancer Center, University of Maryland Baltimore, Baltimore, MD, United States; ^3^ Department of Ophthalmology and Visual Sciences, Marlene and Stewart Greenebaum Comprehensive Cancer, University of Maryland Medical Center, Baltimore, MD, United States; ^4^ Department of Microbiology and Immunology, School of Medicine, University of Maryland Baltimore, Baltimore, MD, United States

**Keywords:** CD27, CD70, immunotherapy, allogeneic hematopoietic cell transplant (alloHCT), graft-*versus*-host disease (GVHD)

## Abstract

Immune checkpoint inhibitor therapies and allogeneic hematopoietic cell transplant (alloHCT) represent two distinct modalities that offer a chance for long-term cure in a diverse array of malignancies and have experienced many breakthroughs in recent years. Herein, we review the CD27-CD70 co-stimulatory pathway and its therapeutic potential in 1) combination with checkpoint inhibitor and other immune therapies and 2) its potential ability to serve as a novel approach in graft-*versus*-host disease (GVHD) prevention. We further review recent advances in the understanding of GVHD as a complex immune phenomenon between donor and host immune systems, particularly in the early stages with mixed chimerism, and potential novel therapeutic approaches to prevent the development of GVHD.

## Introduction

Allogeneic hematopoietic cell transplant (alloHCT) provides the greatest probability for long-term cure in many hematologic malignancies where few other effective therapeutic options exist. However, despite the obvious life-saving benefits of alloHCT, graft-*versus*-host disease (GVHD), a significant toxicity of alloHCT, can be devastating and lead to multi-system tissue damage including the skin, liver, GI tract, and eyes potentially leading to significant morbidity and mortality including liver failure, systemic sclerosis, and severe ocular surface disease (1, 2). The treatment paradigm in alloHCT has evolved rapidly in the last three decades, largely due to a better mechanistic understanding of the complex interactions between donor and host immune cells and host organ systems. This understanding has revolutionized care and dramatically improved patient outcomes. This is well demonstrated by a retrospective analysis comparing alloHCT recipients with grade III and IV acute GVHD from 1997-2006 and 2007-2012 where 12-month treatment related mortality decreased from 58% to 38% in this period of time (3). These improved clinical outcomes have occurred as a result of an improved understanding of the pathogenesis of GVHD. However, despite advances, GVHD remains a significant cause of morbidity and non-relapse related mortality in alloHCT.

The framework of classical acute GVHD occurring in the first 100 days of transplant due to alloreactivity driven by donor T-cells has more recently been supplanted by a more robust understanding involving the intricate interplay of donor and host immune cells with host tissue (4). The initiation phase of GVHD is believed to be mediated by both surviving host and donor Antigen Presenting Cells (APCs) (5, 6). The insult of conditioning chemotherapy and Total Body Irradiation (TBI) has been shown to cause significant changes in hematopoiesis, activation of host APCs, and host tissue damage, leading to an inflammatory environment, which sets the stage for the development of acute GVHD (7–10). This inflammatory milieu includes cytokine release in both hematopoietic and non-hematopoietic compartments, leading to both host and donor T-cell activation and proliferation and alloreactivity which subsequently damages host tissue as GVHD manifests (9–13). A multitude of diverse therapies to alter these underlying mechanisms of GVHD have been adopted into standard clinical practice. As the current standard of care, this has included post-transplant T-cell depletion with cyclophosphamide as well as corticosteroids, calcineurin and Inosine-5′-monophosphate dehydrogenase (IMPDH) inhibitors, and Janus kinase inhibitors; while many others, including checkpoint inhibitors (CPI) and co-stimulatory pathways have also been investigated in GVHD models (6, 14–18).

Another realm of treatment modality in the arena of cancer therapy that has revolutionized the field has been the adoption of CPI therapies, which are now utilized in the treatment of a diverse array of advanced stage malignancies, from non-small cell lung cancer to classical Hodgkin’s lymphoma (19, 20). Despite their successes in a diverse array of malignancies, overall response to CPI therapy remains low, with reported response rates of 12-24% in solid tumors to date (21, 22). The adoption of CPI therapy is based on the premise of the importance of the immune system, particularly the tumor microenvironment and more specifically cytotoxic CD8+ T-cells, in regulating tumor pathogenesis and progression. An important mechanism of tumor immune escape is the attenuation of cytotoxic T-cell activity and proliferation by T-cell exhaustion. Exhaustion occurs by a multifactorial etiology due to persistent tumor antigen exposure, loss of effector cytokine secretion/stimulation [Interleuken-2 (IL-2), Interferon (IFN)-gamma), immunosuppressive cell types (e.g. myeloid derived suppressor cells (MDSCs)], and immunophenotypic changes, including increased checkpoint inhibitor expression [programmed death receptor-1 (PD-1), cytotoxic T-lymphocyte antigen number 4 (CTLA-4), T-cell immunoglobulin mucin-3 (TIM-3), and Lymphocyte-activation gene 3 (LAG-3)] (23, 24).

**Figure 1:**
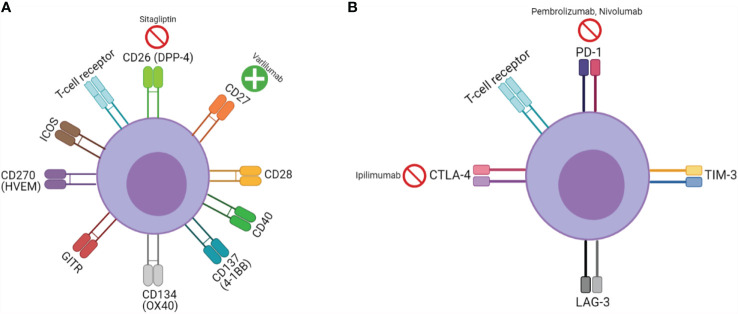
T-cell signaling, function, and pharmacologic targets: **(A)** Co-stimulatory receptors and **(B)** inhibitory receptors; 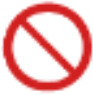
represents inhibitor, 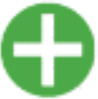
represents agonist.

While CPI targeting agents derive their function by countering an inhibitory signal, an alternate and possibly synergistic approach has been agonizing T-cell stimulatory co-signaling pathways. Co-stimulatory pathways are broadly speaking, either part of the B7/CD28 or tumor necrosis factor (TNF) family (25). Clinically significant co-signaling pathways include CD26, CD27, CD28, CD40, 4-1BB (CD137), OX40 (CD134), glucocorticoid-induced TNF receptor family-related protein (GITR), herpes virus entry mediator (HVEM) (CD270), and inducible T-cell co-stimulator (ICOS) (26–29). Although a significant oversimplification, this is analogously described as CPI therapy being akin to “pulling the foot off of the brake”, while agonizing co-signaling pathways are “pressing down on the accelerator” (See **Figure 1**).

Thus far, the clinical use of co-stimulatory signaling pathways have lagged behind that of CPIs. However, given the need for improved response rates in those undergoing CPI therapy, the use of co-stimulatory pathways has been explored as a potential therapeutic intervention to increase responses. Additionally, the co-stimulatory receptors CD28 and 4-1BB (CD137) have been utilized in the development of both experimental and commercially available second generation chimeric antigen receptor T-cell (CAR-T) therapies leading to significantly greater activation, expansion, and persistence of CAR-T cells (30, 31). More recently, these pathways have also been studied and exploited as potential therapeutic targets for attenuating GVHD. Ultimately, however, the concern remains that any immunosuppressive GVHD-targeted therapy may adversely impact the graft-*versus*-tumor (GVT) effect as there is a strong correlation between incidence and severity of GVHD and disease free survival (32).

Thus, it is critical to identify co-stimulatory pathways which when blocked decrease GVHD but do not interfere with GVT. One potential way to decrease the incidence of GVHD would be by inhibiting a co-stimulatory receptor thereby attenuating CD4+ and CD8+ cytotoxic T-cell activity. CD26 has been studied in both pre-clinical and clinical models, while the CD27-CD70 pathway has been studied extensively in pre-clinical murine models. In murine models, inhibition of CD26 [also known as dipeptidyl peptidase-4 (DPP4)] by a monoclonal antibody has been demonstrated to decrease GVHD incidence without compromising GVT (33). In a small, non-randomized clinical trial, the diabetic medication and DPP4 inhibitor, sitagliptin, was administered from day -1 to day +14 of alloHCT, resulting in a low incidence (5%) of grades II-IV GVHD followed to day +100 (34). CD27-CD70 has also been studied in murine and cellular models. Cao et al. and colleagues demonstrated that antagonism of the host CD27-CD70 co-stimulatory pathway significantly increased, rather than decreased, the development of murine GVHD (35, 36).

Herein, we conduct an in-depth review of the CD27-CD70 pathway and its application in both GVHD attenuation following alloHCT and its use in the treatment of numerous malignancies in combination with CPI therapies.

## CD27-CD70 Pathway

CD27, a member of the TNF receptor superfamily is constitutively expressed on naive T-cells, memory B-cells, NK-cells, and hematopoietic stem cells (HSCs) and progenitor cells (37–40). CD27 is a transmembrane phosphoglycoprotein expressed on both CD4+ and CD8+ T-cells with increased expression upon T-cell activation and shedding from the cellular surface and formation of soluble CD27 (sCD27) upon activation (41, 42). CD70 (CD27L), the only ligand for CD27, is a tightly regulated transmembrane glycoprotein expressed on both B and T-lymphocytes and APCs (43). CD70 has structural similarity to other TNF superfamily members (TNFα, FasL, receptor activator of NF-κB ligand (RANKL), TNF-related apoptosis-inducing ligand (TRAIL), 4-1BBL, CD30L, and CD40L) (44). Upon binding of CD70, CD27 is bound to TNF receptor-associated factors (TRAFs) leading to intracellular signaling which potentiates survival and activation of T, B, and natural killer (NK) cells *via* Traf2 and Traf5 signaling and activation of the NF-κB pathway (45). The interaction of CD27-CD70 is tightly regulated to prevent overexpression and subsequent excessive lymphocyte activation. In a normal physiologic state, CD70 is only expressed in the thymus and lamina propria (46). However, stimulation by interaction with toll-like receptor (TLR) ligands and dendritic cells (DCs), the most prominent of APCs, results in increased expression of CD70 on DCs, albeit transiently (47). Although exceedingly rare, human CD27 deficiency has been associated with Epstein-Barr virus (EBV) associated lymphoproliferative disorders [lymphoma and hemophagocytic lymphohistiocytosis (HLH)], and recurrent infections (48, 49).

Under pro-inflammatory conditions (infection, malignancy, autoimmune conditions) CD27-CD70 activity is increased, leading to proliferation and survival of lymphocytes with multiple downstream effects (50). CD27-CD70 signaling has also been shown to promote B-cell activation and terminal differentiation to plasma cells, increase cytotoxic CD8+ T-cell activity, promote TNFα production by T-cells, and increase NK-cell activity with production of IFNγ and IL-2 (44). In response to IFN-γ secretion due to CD27-CD70 stimulation, C-X-C motif chemokine ligand 10 (CXCL10) [also known as interferon gamma-induced protein 10 (IP-10)] has been demonstrated to increase the CD8+ T-cell effector pool (51). Additionally, CD27 expression was noted in a subset of IFNγ producing γδ T-cells following infection, while CD27 negative γδ T-cells did not produce IFNγ, suggesting a role for CD27 in regulation of interferon and specific cytokine production in immune responses (52). The CD27 co-stimulatory response has also been shown to be key for acute effector CD8+ T-cell expression of IL-7Rα, an important cytokine for the generation of CD8+ T-memory cells (53).

In the bone marrow, HSCs are a heterogeneous population serving as precursors to all myeloid and lymphoid lineage cell types (54). In contrast to their mature counterparts, HSCs have limited surface antigen expression and lack lineage specific cell surface markers. However, interestingly, HSCs have been shown to exhibit high CD27 expression (90% of HSCs in murine models express CD27) (38, 55). In murine *in vitro* models, CD27 agonism of bone marrow progenitor cells decreased monocytic differentiation and overall inhibited leukocyte differentiation, while in competitive transplantation assays CD27 agonism decreased donor B and T lymphocytes, suggesting the CD27-CD70 pathway’s ability to influence hematopoiesis and immune cell differentiation (56).

## CD27-CD70 for Cancer Immunotherapy

At the time of writing, the study of the CD27-CD70 pathway in GVHD remains confined to murine and cellular models, with ongoing studies seeking to better understand the effect of CD27 agonism on donor hematopoietic cell differentiation, engraftment, and GVT effect. However, a CD27 agonizing monoclonal antibody, varlilumab, has been extensively studied both in *in vitro* and *in vivo* in phase I/II clinical trials for a number of hematologic and solid tumor types, including Hodgkin’s lymphoma, non-Hodgkin’s lymphoma (NHL), glioblastoma, melanoma, renal cell carcinoma, prostate adenocarcinoma, colorectal adenocarcinoma, and ovarian cancer (57–60). (See **Table 1** for further details of previous and ongoing registered clinical trials.) The rationale behind these trials has been to study the impact of CD27 agonism alone as a T-cell co-stimulator as well as to determine if it functions in a synergistic manner in combination with checkpoint inhibitor therapy and cancer vaccines to improve antineoplastic response. Additionally, many B-cell lymphomas express CD27, which may serve as a direct target in a fashion similar to CD20 targeting with Rituximab. In multiple *in vitro* and murine tumor models, PD1/PDL1 blockade in combination with an agonist CD27 monoclonal antibody was shown to enhance CD8+ cytotoxic T-cell expansion and function in an IL-2 dependent manner with gene expression changes promoting T-cell proliferation (66). In various syngeneic tumor murine models, varlilumab was shown to have two predominating anti-tumor mechanisms of action by its co-stimulatory effect and Treg depletion (67).

**Table 1:** Clinical trials with CD27 agonizing monoclonal antibody.

Study Title:	Trial identifier:	Status:	Sponsor:	Phase:	Conditions:	Intervention:	Results^+^:	Adverse Events^++^:	Related Publications:
A Dose Escalation and Cohort Expansion Study of Anti-CD27 (Varlilumab) and Anti-PD-1 (Nivolumab) in Advanced Refractory Solid Tumors	NCT02335918	Completed	Celldex Therapeutics	I/II	Squamous Cell Carcinoma of the Head and Neck, Ovarian Carcinoma, Colorectal Cancer, Renal Cell Carcinoma, Glioblastoma multiforme	varlilumab and nivolumab	Colorectal cancer- 2/41 patients PR, 7/41 patients SD Ovarian cancer- 5/49 patients PR, 19/49 patients SD Squamous Cell of the Head and Neck- 1/3 patients PR	Colorectal cancer- 3/42 patients with mixed motor sensory neuropathy, pneumonitis, elevated ALT) Ovarian cancer- 2/66 patients with acute kidney injury, hepatitis, small bowel obstruction	(61, 62)
A Study of CDX-1127 (Varlilumab) in Patients With Select Solid Tumor Types or Hematologic Cancers	NCT01460134	Completed	Celldex Therapeutics	I	CD27 Expressing B-cell Malignancies (Hodgkin’s Lymphoma, Chronic Lymphocytic Leukemia, Mantle Cell Lymphoma, Marginal Zone B Cell Lymphoma, Any T-cell Malignancy, Solid Tumors (Metastatic Melanoma, Renal (Clear) Cell Carcinoma, Hormone-refractory Prostate Adenocarcinoma, Ovarian Cancer, Colorectal Adenocarcinoma, Non-small Cell Lung Cancer), Burkett’s Lymphoma, Primary Lymphoma of the Central Nervous System	CDX-1127 (varlilumab)	Hodgkin’s Lymphoma- 1/10 patients CR, 1/10 patients with SD Non-Hodgkin Lymphoma- 3/18 patients SD	Any adverse event-9/34 patients Grade 2 cytopenias- 3/34 patients Grade 2 fatigue- 5/34 patients Grade 2 neurologic symptoms- 2/6 Grade 2 hypotension- 1/34 patients	(57)
Study of ONT-10 and Varlilumab to Treat Advanced Ovarian or Breast Cancer	NCT02270372	Completed	Cascadian Therapeutics Inc.	I	Advanced Breast Carcinoma, Advanced Ovarian Carcinoma	ONT-10 and varlilumab	None Posted	None Posted	None Posted
A Study of Varlilumab and IMA950 Vaccine Plus Poly-ICLC in Patients With WHO Grade II Low-Grade Glioma (LGG)	NCT02924038	Recruiting	Nicholas Butowski, MD,University of California San Fransisco	I	Glioma, Malignant Glioma, Astrocytoma, Grade II, Oligodendroglioma, Glioma, Astrocytic, Oligoastrocytoma, Mixed	IMA950 vaccine, poly-ICLC vaccine, and varlilumab	None Posted	None Posted	None Posted
Nivolumab With or Without Varlilumab in Treating Patients With Relapsed or Refractory Aggressive B-cell Lymphomas	NCT03038672	Recruiting	National Cancer Institute	II	Numerous subtypes of Non-Hodgkin lymphoma	varlilumab and nivolumab	None Posted	None Posted	(63)
A Combination of Rituximab and Varlilumab Immunotherapy in Patients With B-cell Lymphoma (RIVA)	NCT03307746	Recruiting	University Hospital Southampton NHS Foundation Trust	I/II	CD20+ B-Cell Lymphoma	varlilumab and rituximab	None Posted	None Posted	(64)
Atezolizumab and Varlilumab in Combination With Radiation Therapy for NSCLC	NCT04081688	Recruiting	Rutgers, The State University of New Jersey	I	Refractory Lung Non-Small Cell Carcinoma, Stage IV Lung Cancer	varlilumab, atezolizumab, and stereotactic radiation therapy	None Posted	None Posted	None Posted
Vaccination With 6MHP, With or Without Systemic CDX-1127, in Patients With Stage II-IV Melanoma	NCT03617328	Recruiting	Craig L Slingluff, Jr MD,University of Virginia	I/II	Melanoma	CDX-1127 (varlilumab), 6MHP, Montanide ISA-51, polyICLC	None Posted	None Posted	None Posted
DC Migration Study to Evaluate TReg Depletion In GBM Patients With and Without Varlilumab (DERIVE)	NCT03688178	Recruiting	Gary Archer Ph.D., Duke University	II	Glioblastoma	Human CMV pp65-LAMP mRNA-pulsed autologous DCs, temozolomide, varlilumab, Td, 111In-labeled DCs, Unpulsed DCs	None Posted	None Posted	None Posted
A Study of Varlilumab (Anti-CD27) and Ipilimumab and CDX-1401 in Patients With Unresectable Stage III or IV Melanoma	NCT02413827	Terminated	Celldex Therapeutics	I/II	Unresectable Stage III or Stage IV Melanoma	varlilumab and ipilimumab; varlilumab, ipilimumab, CDX-1401, and poly-ICLC	None Posted	None Posted	None Posted
A Study of Varlilumab (Anti-CD27) and Sunitinib in Patients With Metastatic Clear Cell Renal Cell Carcinoma	NCT02386111	Terminated	Celldex Therapeutics	I	Carcinoma, Renal Cell, Urogenital/Urologic Neoplasms	varlilumab and sunitinib	None Posted	None Posted	None Posted
A Study of Varlilumab and Atezolizumab in Patients With Advanced Cancer	NCT02543645	Terminated	Celldex Therapeutics	I/II	Carcinoma, Renal Cell, Urogenital/Urologic Neoplasms, Melanoma, Triple negative breast cancer, Head and neck cancer, Non-small cell lung cancer	varlilumab and atezolizumab	None Posted	None Posted	None Posted
Pilot Study of SBRT and CDX-1127 in Prostate Cancer (Prostate-04)	NCT02284971	Terminated	James Larner, MD, University of Virginia	I	Prostate cancer	Stereotactic Body Radiation and varlilumab	None Posted	None Posted	None Posted
A Study of Glembatumumab Vedotin as Monotherapy or in Combination With Immunotherapies in Patients With Advanced Melanoma	NCT02302339	Terminated	Celldex Therapeutics	II	Melanoma	glembatumumab vedotin, glembatumumab vedotin and varlilumab, glembatumumab vedotin and PD-1 targeted checkpoint inhibitor, gembatumumab vedotin and CDX-301	1/31 patients with objective response in glembatumumab vedotin and varlilumab group	14/34 with serious adverse event reported in glembatumumab vedotin and varlilumab group	(65)

Results generated from search for “CD27 antibody” and “varlilumab.” Publications listed by google scholar, PubMed, and clinicaltrials.gov reported publications.

+CR, Complete Response; PR, Partial Response; SD, Stable Disease; PD, Progressive disease per RECIST 1.1 criteria.

++ Adverse events graded per National Cancer Institute–issued Common Terminology Criteria for Adverse Events version 4.0.

The recent development of a bispecific antibody, CDX-527, has sought to improve the efficacy of the CD27 agonism and PD1/PDL1 blockade by combining CD27 agonism with cross-linking through PDL1 and Fc receptors (68). CDX-527 was demonstrated to have potent T-cell activation by increasing IL-2 and IFNγ production and anti-tumor activity to CD27-expressing lymphoma cells in an immunodeficient mouse model, with comparable anti-tumor activity to separate CD27 agonizing and PDL1 inhibiting monoclonal antibodies. Similarly, a hexavalent TNF receptor agonist (HERA) targeting CD27 has been developed and demonstrated to cause an increased proliferative response to CD4+ and CD8+ T-cells when compared to CD27L *in vitro* with healthy human T-cells and *in vivo* in murine models (69).

In addition to combination with checkpoint inhibitor therapy, the combination of anti-CD20 and CD27 agonizing monoclonal antibodies has been investigated in an immunocompetent murine B-cell lymphoma and B-chronic lymphocytic leukemia models with a 100% tumor remission rate noted at 100 days (70). The combination antibody group was noted to have significantly increased CD8+ cytotoxic T-cells and Treg cells compared to CD20 monoclonal antibody alone. Additionally, the combination was shown to promote tumor infiltration and activation of myeloid cells and macrophages towards an anti-tumor phenotype. The efficacy of this combined therapy is currently being investigated in humans in the RIVA study, a phase IIa open-label clinical trial of patients with relapsed/refractory CD20+ B-cell lymphomas (64).

In the limited clinical trials to date, the CD27 agonizing monoclonal antibody, varlilumab, as monotherapy and with PD1/PDL1 checkpoint inhibitor therapy (nivolumab, atezolizumab), has resulted in varying degrees of objective clinical responses in a subset of cancer patients enrolled. This has included complete remission in Hodgkin’s lymphoma and partial responses in ovarian, colorectal, and squamous cell cancer of the head and neck (see **Table 1**). Furthermore, it was well tolerated with limited, predominately grade 1-2 toxicities (fatigue, nausea, and thrombocytopenia) reported at all dose levels up to 10mg/kg in trial subjects (57, 71). In ovarian cancer patients, the combination therapy of varlilumab and nivolumab resulted in increased tumor expression of PD-L1 and CD8+ tumor infiltrating lymphocytes in 61% and 58% of patients, respectively (61). Upon administration to trial subjects, soluble CD27 plasma concentrations were significantly increased in a dose-dependent fashion. Cytokines were also increased in a dose-independent manner, indicative of an inflammatory response, particularly IL-12, monokine induced by IFNγ (CXCL9), MIP-1β (CCL4), and monocyte chemoattractant protein-1 (CCL2). In *in vitro* studies of T-cell isolates from healthy volunteer peripheral blood mononuclear cells (PBMCs) treated with varlilumab revealed that both CD4+ and CD8+ T-cells were stimulated (although with a greater emphasis on CD8+ activation), which was accompanied by upregulation of other co-stimulatory pathways (4-1BB, OX40, GITR, and ICOS) along with the inhibitory PD1 pathway (72).

CD27 agonism alone and with an PD1 checkpoint inhibitor has also been explored as a potential mechanism of increasing the efficacy of tumor-specific peptide vaccines by enhancing CD4+ helper T-cell and CD8+ cytotoxic T-cell response following vaccination (73, 74). Clinical trials are currently underway combining varlilumab with 6MHP, a vaccine of six melanoma peptides; ONT-10, a peptide vaccine incorporating MUC1 tumor antigen, a TLR-4 agonist, and PET lipid A in breast and ovarian malignancies; and IMA950, a multi-peptide vaccine with 11 glioma-associated antigens.

While varlilumab has yet to obtain an FDA indicated approval for use, six clinical trials with varlilumab are actively recruiting patients with B and T-cell lymphomas, neurologic malignancies, melanoma, and non-small cell lung cancer (**Table 1**).

## CD27-CD70 in alloHCT and GVHD

**Figure 2:**
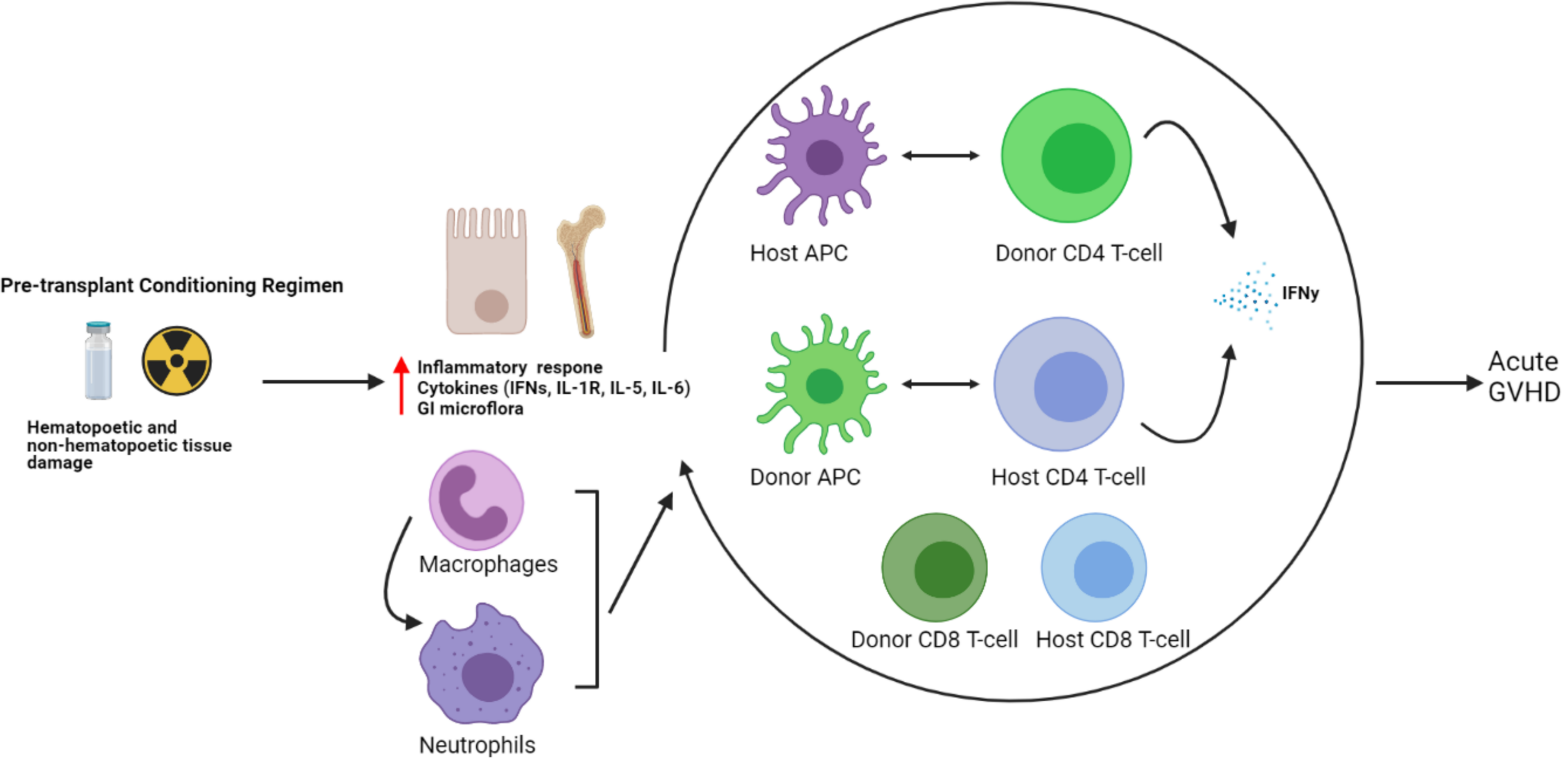
Host and donor immune cell interactions and pathogenesis of acute GVHD. The current understanding of acute GVHD pathogenesis involves a complex interaction of host and donor immune cells.

Traditionally, the prevailing thought behind the etiology of GVHD rested solely with donor immune cells, particularly T-cells becoming activated upon alloreactivity to host antigens. However, more recently, the complex interaction between donor and host immune systems leading to GVHD has been noted, particularly in the early stages of alloHCT, where a mixed chimerism exists (75, 76). While the pre-alloHCT conditioning regimen clears the peripheral blood of most host T-cells, they often persist for many months in the tissues most effected by acute GVHD—the skin and gastrointestinal tract. The role of persistent host T-cells mediating acute GVHD by interaction with donor APCs has been noted in murine models and in alloHCT transplant patients with increased IFNγ–secreting CD4+ T-cells in skin GVHD biopsies compared to healthy controls, as well as an increased monocyte population with upregulation of chemoattractant receptors and IFN-response genes (IFITM1 and GBP1) compared with healthy controls (77). Conversely, the interaction between host APCs and donor T-cells had been reported earlier to be associated with the development of acute GVHD (7, 11). These findings underscore the complexity of immune interactions between a diverse array of both donor and host immune cells that may ultimately result in the development of GVHD (see **Figure 2**).

The most commonly employed conditioning regimens in alloHCT are given with myeloablative or reduced intensity/non-myeloablative intensity consisting of a combination of myelotoxic chemotherapeutic agents with or without TBI (78–80). The conditioning regimen acts as a profound insult to the marrow microenvironment leading to increased cytokine and interferon levels. This also impacts the function of HSCs, akin to emergency hematopoiesis seen in other stressful states such as severe infection and radiation exposure where pro-inflammatory signals (IFNα/β, IFNγ, TNFα, IL1-R, IL-5, and IL-6) encourage HSC response and subsequent downstream maturation and differentiation (10, 12, 13). In a study of the bone marrow microenvironment in 28 patients undergoing alloHCT for hematologic malignancies, dramatic changes were noted over the course of one year. In six patients undergoing a myeloablative conditioning regimen, bone marrow samples were obtained on the day of transplantation (day 0) to determine the effect of conditioning, which demonstrated a statistically significant increase in Tregs and a 30-fold increase in IFNγ concentration (9). However, the concentration of IL-2, IL-6, IL-10, and IL-17A were not significantly different, while IL-1b, IL-4, IL-11, and TNFα were mostly undetectable. By day +100 (the timeframe for classical acute GVHD), the percentage of Tregs and concentration of IFNγ was comparable to healthy donors, suggesting a normalization of the bone marrow microenvironment by day +100.

Collectively, these findings suggest the importance of alterations in the bone marrow microenvironment following the noxious insult of the conditioning regimen leading to emergency hematopoiesis and the complex interaction of host and donor immune cells which may persist for many months following alloHCT, during the time acute GVHD is most likely to occur.

**Figure 3:**
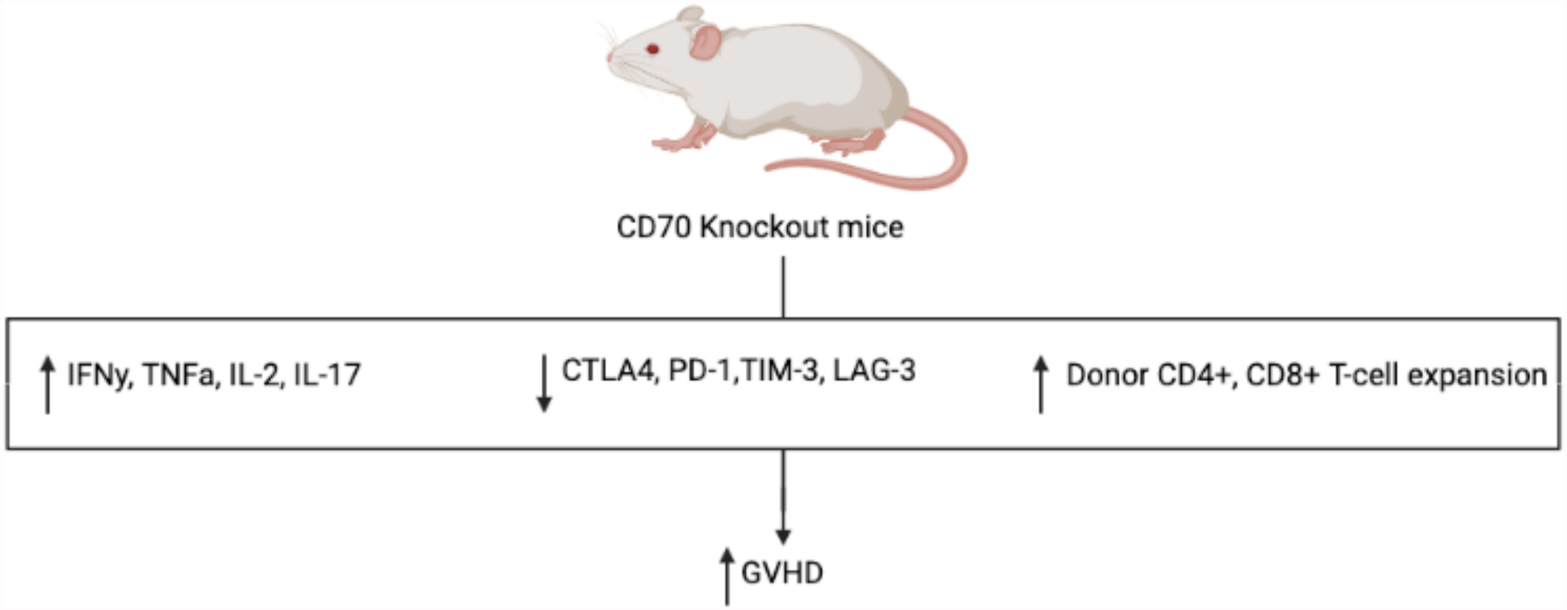
Mechanism of increased GVHD in CD70 knockout mice. Compared to WT control, CD70 knockout mice have significantly more GVHD with increased inflammatory cytokines, decreased CPI expression, and increased expansion of donor T-cells.

Given its ability to broadly influence hematopoietic differentiation and lymphocyte activity, the CD27-CD70 pathway presents itself as an attractive and novel target in the development of a future GVHD targeted therapy. Similar to the inhibition of CD26, it has been hypothesized that inhibition of CD27 would result in attenuated GVHD, namely by decreasing cytotoxic T-cell alloreactivity. However, in murine models, the administration of an anti-CD70 monoclonal antibody following alloHCT resulted in significantly increased GVHD in a dose dependent fashion (35). This was an unexpected finding, suggesting an alternative and more vital mechanism relating to the pathogenesis and development of GVHD. In further study, while APC-expressed CD70 provides a co-stimulatory signal, T-cell-expressed CD70 serves an inhibitory role in T-cell response, akin to CPIs PD-1 and TIM-3, leading to decreased inflammatory response and GVHD in murine models (36). To better elucidate the mechanism of the CD27-CD70 pathway and its impact on GVHD pathogenesis, cytokines associated with GVHD were measured in CD70 knockout host mice which showed significantly higher levels of pro-inflammatory IFNγ, TNFα, IL-2, and IL-17 when compared to WT mice (see **Figure 3**) (35). This was noted to result in significant changes in host and donor immunophenotype with expansion of donor, but not host, CD4+ and CD8+ T-cells. Furthermore, CD70 knockout was studied in host hematopoietic and non-hematopoietic compartments, with CD70 knockout in hematopoietic compartments shown to result in greater GVHD, indicating that CD70 expression in host hematopoietic cells was the main contributor to the development of GVHD in these models. Meanwhile, interestingly, T-cell derived CD70 was shown to have an inhibitory role by inhibiting allogeneic CD4+ and CD8+ T-cell responses *via* caspase-dependent T-cell apoptosis and upregulation of inhibitory immune checkpoint inhibitor pathways (36). Thus, based on these findings, the CD27-CD70 pathway has multiple immunomodulating effects, both activating and inactivating, depending on the environment and cell type expressing CD27 or CD70. This further suggests that the CD27-CD70 pathway also has an impact on host hematopoiesis and immune cell differentiation, impacting the development of GVHD, perhaps by promoting a decrease in inflammatory cell types in favor of less inflammatory ones, although more studies are required to develop an understanding of the underlying mechanisms.

## Concluding Remarks

More recently, with the evolution of CPI and other T-cell concentrated therapies in other fields of Oncology, co-stimulatory mechanisms involved in the activation and proliferation of T-cells have been explored. Of notable importance, agonism of the co-stimulatory CD27-CD70 pathway, a member of the TNF superfamily, has been studied as a potential therapeutic intervention as an oncologic therapy for multiple tumor cell types as well as a therapeutic intervention to attenuate GVHD. Thus, agonism of the CD27-CD70 pathway presents itself as a novel future therapeutic target, particularly with the availability of a CD27 agonizing monoclonal antibody that has completed phase I/II study and been shown to be quite safe and well tolerated with minimal high-grade toxicities reported.

## Author Contributions

All authors listed have made a substantial, direct, and intellectual contribution to the work and approved it for publication.

## Funding

5T32CA154274-10, University of Maryland School of Medicine, Oncology Fellow Research Grant (FL), R01HL135325 (XC).

## Conflict of Interest

The authors declare that the research was conducted in the absence of any commercial or financial relationships that could be construed as a potential conflict of interest.

## Publisher’s Note

All claims expressed in this article are solely those of the authors and do not necessarily represent those of their affiliated organizations, or those of the publisher, the editors and the reviewers. Any product that may be evaluated in this article, or claim that may be made by its manufacturer, is not guaranteed or endorsed by the publisher.

## References

[1] Cooke KR Luznik L Sarantopoulos S Hakim FT Jagasia M Fowler DH. The Biology of Chronic Graft-*Versus*-Host Disease: A Task Force Report From the National Institutes of Health Consensus Development Project on Criteria for Clinical Trials in Chronic Graft-*Versus*-Host Disease. Biol Blood Marrow Transplant (2017). doi: 10.1016/j.bbmt.2016.09.023 PMC6020045 27713092

[2] Inamoto Y Valdés-Sanz N Ogawa Y Alves M Berchicci L Galvin J. Ocular Graft-*Versus*-Host Disease After Hematopoietic Cell Transplantation: Expert Review From the Late Effects and Quality of Life Working Committee of the CIBMTR and Transplant Complications Working Party of the EBMT. Bone Marrow Transplant (2019) 54:662–73. doi: 10.1038/s41409-018-0340-0 30531954

[3] El-Jawahri A Li S Antin JH Spitzer TR Armand PA Koreth J. Improved Treatment-Related Mortality and Overall Survival of Patients With Grade IV Acute GVHD in the Modern Years. Biol Blood Marrow Transplant (2016) 22:910–8. doi: 10.1016/j.bbmt.2015.12.024 26748160

[4] Holtan SG Marcelo P Weisdorf DJ. Acute Graft-*Versus*-Host Disease: A Bench-to-Bedside Update. Blood (2014) 124:363–73. doi: 10.1182/blood-2014-01-514786 PMC4102709 24914140

[5] Duffner UA Maeda Y Cooke KR Reddy P Ordemann R Liu C. Host Dendritic Cells Alone Are Sufficient to Initiate Acute Graft-*Versus*-Host Disease. J Immunol (2004) 172:7393–8. doi: 10.4049/jimmunol.172.12.7393 15187116

[6] Koyama M Hill GR. Alloantigen Presentation and Graft-*Versus*-Host Disease: Fuel for the Fire. Blood (2016) 127:2963–70. doi: 10.1182/blood-2016-02-697250 27030390

[7] Zhang Y Louboutin J-P Zhu J Rivera AJ Emerson SG. Preterminal Host Dendritic Cells in Irradiated Mice Prime CD8+ T Cell–Mediated Acute Graft-*Versus*-Host Disease. J Clin Invest (2002) 109:1335–44. doi: 10.1172/JCI0214989 PMC150980 12021249

[8] Saada JI Pinchuk IV Barrera CA Adegboyega PA Suarez G Mifflin RC. Subepithelial Myofibroblasts are Novel Nonprofessional APCs in the Human Colonic Mucosa. J Immunol (2006) 177:5968–79. doi: 10.4049/jimmunol.177.9.5968 17056521

[9] Ciomber A Mitrus I Fidyk W Smagur A Chwieduk A Glowala-Kosinska M. Immunological Properties of Bone Marrow Microenvironment 1 Year After Allogeneic Hematopoietic Stem Cell Transplantation. Exp Hematol (2016) 44:1172–1180.e1. doi: 10.1016/j.exphem.2016.08.001 27524270

[10] Croker BA Silke J Gerlic M. Fight or Flight: Regulation of Emergency Hematopoiesis by Pyroptosis and Necroptosis. Curr Opin Hematol (2015) 22:293–301. doi: 10.1097/MOH.0000000000000148 26049749 PMC4459526

[11] Shlomchik WD Couzens MS Tang CB McNiff J Robert ME Liu J. Prevention of Graft *Versus* Host Disease by Inactivation of Host Antigen- Presenting Cells. Sci (80- ) (1999) 285:412–5. doi: 10.1126/science.285.5426.412 10411505

[12] Pucella JN Upadhaya S Reizis B. The Source and Dynamics of Adult Hematopoiesis: Insights From Lineage Tracing. Annu Rev Cell Dev Biol (2020) 36:529–50. doi: 10.1146/annurev-cellbio-020520-114601 32580566

[13] Linz BM Brickey WJ Ting JP Cairns BA Maile R. NLRP12 Limits Tnfα-Induced Apoptosis of Monocyte Progenitor Cells During Emergency Hematopoiesis. J Immunol (2016) 196:62.13 LP–62.13.

[14] O’Donnell PV Luznik L Jones RJ Vogelsang GB Leffell MS Phelps M. Nonmyeloablative Bone Marrow Transplantation From Partially HLA-Mismatched Related Donors Using Posttransplantation Cyclophosphamide. Biol Blood Marrow Transplant (2002) 8:377–86. doi: 10.1053/bbmt.2002.v8.pm12171484 12171484

[15] Jacobson P Uberti J Davis W Ratanatharathorn V. Tacrolimus: A New Agent for the Prevention of Graft-*Versus*-Host Disease in Hematopoietic Stem Cell Transplantation. Bone Marrow Transplant (1998) 22:217–25. doi: 10.1038/sj.bmt.1701331 9720734

[16] Baudard M Vincent A Moreau P Kergueris MF Harousseau JL Milpied N. Mycophenolate Mofetil for the Treatment of Acute and Chronic GVHD Is Effective and Well Tolerated But Induces a High Risk of Infectious Complications: A Series of 21 BM or PBSC Transplant Patients. Bone Marrow Transplant (2002) 30:287–95. doi: 10.1038/sj.bmt.1703633 12209350

[17] Escamilla Gómez V García-Gutiérrez V López Corral L García Cadenas I Pérez Martínez A Márquez Malaver FJ. Ruxolitinib in Refractory Acute and Chronic Graft-*Versus*-Host Disease: A Multicenter Survey Study. Bone Marrow Transplant (2020) 55:641–8. doi: 10.1038/s41409-019-0731-x PMC7051903 31700138

[18] Zeiser R von Bubnoff N Butler J Mohty M Niederwieser D Or R. Ruxolitinib for Glucocorticoid-Refractory Acute Graft-*Versus*-Host Disease. N Engl J Med (2020) 382:1800–10. doi: 10.1056/NEJMoa1917635 32320566

[19] KEYTRUDA HIGHLIGHTS OF PRESCRIBING INFORMATION. FDA Package Insert (2014). Available at: https://www.accessdata.fda.gov/drugsatfda_docs/label/2020/125514s066lbl.pdf.

[20] Kuruvilla J Ramchandren R Santoro A Paszkiewicz-Kozik E Gasiorowski R Johnson N. KEYNOTE-204: Randomized, Open-Label, Phase III Study of Pembrolizumab (Pembro) *Versus* Brentuximab Vedotin (BV) in Relapsed or Refractory Classic Hodgkin Lymphoma (R/R cHL). J Clin Oncol (2020) 38:8005. doi: 10.1200/JCO.2020.38.15_suppl.8005 33721562

[21] Haslam A Prasad V. Estimation of the Percentage of US Patients With Cancer Who Are Eligible for and Respond to Checkpoint Inhibitor Immunotherapy Drugs. JAMA Netw Open (2019) 2:e192535–e192535. doi: 10.1001/jamanetworkopen.2019.2535 31050774 PMC6503493

[22] Webster RM. The Immune Checkpoint Inhibitors: Where are We Now? Nat Rev Drug Discovery (2014) 13:883–4. doi: 10.1038/nrd4476 25345674

[23] Catakovic K Klieser E Neureiter D Geisberger R. T Cell Exhaustion: From Pathophysiological Basics to Tumor Immunotherapy. Cell Commun Signal (2017) 15:1. doi: 10.1186/s12964-016-0160-z 28073373 PMC5225559

[24] Tesi RJ. MDSC; the Most Important Cell You Have Never Heard of. Trends Pharmacol Sci (2019) 40:4–7. doi: 10.1016/j.tips.2018.10.008 30527590

[25] Mir MA. Costimulation in Lymphomas and Cancers. In: Mir MABT-D, editor. Developing Costimulatory Molecules for Immunotherapy of Diseases. Waltham, MA, USA: Academic Press (2015). p. 185–254. doi: 10.1016/b978-0-12-802585-7.00005-4

[26] Nandi D Pathak S Verma T Singh M Chattopadhyay A Thakur S. T Cell Costimulation, Checkpoint Inhibitors and Anti-Tumor Therapy. J Biosci (2020) 45:50. doi: 10.1007/s12038-020-0020-2 32345776

[27] Hendriks J Xiao Y Borst J. CD27 Promotes Survival of Activated T Cells and Complements CD28 in Generation and Establishment of the Effector T Cell Pool. J Exp Med (2003) 198:1369–80. doi: 10.1084/jem.20030916 PMC2194245 14581610

[28] Ronchetti S Nocentini G Petrillo MG Riccardi C. CD8+ T Cells: GITR Matters. ScientificWorldJournal (2012) 2012:308265. doi: 10.1100/2012/308265 22654588 PMC3361162

[29] Hatano R Ohnuma K Yamamoto J Dang NH Morimoto C. CD26-Mediated Co-Stimulation in Human CD8+ T Cells Provokes Effector Function via Pro-Inflammatory Cytokine Production. Immunology (2013) 138:165–72. doi: 10.1111/imm.12028 PMC3575769 23113658

[30] Jain MD Bachmeier CA Phuoc VH Chavez JC. Axicabtagene Ciloleucel (KTE-C19), an Anti-CD19 CAR T Therapy for the Treatment of Relapsed/Refractory Aggressive B-Cell non-Hodgkin’s Lymphoma. Ther Clin Risk Manage (2018) 14:1007–17. doi: 10.2147/TCRM.S145039 PMC5987753 29910620

[31] Weinkove R George P Dasyam N McLellan AD. Selecting Costimulatory Domains for Chimeric Antigen Receptors: Functional and Clinical Considerations. Clin Transl Immunol (2019) 8:e1049. doi: 10.1002/cti2.1049 PMC6511336 31110702

[32] Horowitz MM Gale RP Sondel PM Goldman JM Kersey J Kolb HJ. Graft-*Versus*-Leukemia Reactions After Bone Marrow Transplantation. Blood (1990) 75:555–62. doi: 10.1182/blood.V75.3.555.555 2297567

[33] Hatano R Ohnuma K Yamamoto J Dang NH Yamada T Morimoto C. Prevention of Acute Graft-*Versus*-Host Disease by Humanized Anti-CD26 Monoclonal Antibody. Br J Haematol (2013) 162:263–77. doi: 10.1111/bjh.12378 23692598

[34] Martin PJ. Sitagliptin to Prevent Acute Graft-*Versus*-Host Disease. N Engl J Med (2021) 384:70–1. doi: 10.1056/NEJMe2032581 33406333

[35] Leigh ND O’Neill RE Du W Chen C Qiu J Ashwell J. Host-Derived CD70 Suppresses Murine Graft-*Versus*-Host Disease by Limiting Donor T Cell Expansion and Effector Function. J Immunol (2017) 199:336–47. doi: 10.4049/jimmunol.1502181 PMC5503479 28550198

[36] O’Neill RE Du W Mohammadpour H Alqassim E Qiu J Chen G. T Cell–Derived CD70 Delivers an Immune Checkpoint Function in Inflammatory T Cell Responses. J Immunol (2017) 199:3700–7310. doi: 10.4049/jimmunol.1700380 29046346 PMC5687300

[37] Maurer D Holter W Majdic O Fischer GF Knapp W. CD27 Expression by a Distinct Subpopulation of Human B Lymphocytes. Eur J Immunol (1990) 20:2679–84. doi: 10.1002/eji.1830201223 1702722

[38] Wiesmann A Phillips RL Mojica M Pierce LJ Searles AE Spangrude GJ. Expression of CD27 on Murine Hematopoietic Stem and Progenitor Cells. Immunity (2000) 12:193–9. doi: 10.1016/S1074-7613(00)80172-7 10714685

[39] Denoeud J Moser M. Role of CD27/CD70 Pathway of Activation in Immunity and Tolerance. J Leukoc Biol (2011) 89:195–203. doi: 10.1189/jlb.0610351 20699361

[40] Takeda K Oshima H Hayakawa Y Akiba H Atsuta M Kobata T. CD27-Mediated Activation of Murine NK Cells. J Immunol (2000) 164:1741–5. doi: 10.4049/jimmunol.164.4.1741 10657619

[41] Mak TW Saunders ME. 14 - T Cell Activation. Mak TW Saunders M. E. B. T.-T. I. R., editors. Burlington, MA, USA: Academic Press (2006) p. 373–401. doi: 10.1016/B978-012088451-3.50016-8

[42] Hintzen RQ van Lier RAW Kuijpers KC Baars PA Schaasberg W Lucas CJ. Elevated Levels of a Soluble Form of the T Cell Activation Antigen CD27 in Cerebrospinal Fluid of Multiple Sclerosis Patients. J Neuroimmunol (1991) 35:211–7. doi: 10.1016/0165-5728(91)90175-7 1659587

[43] Van Nieuwenhuijze A Liston A. The Molecular Control of Regulatory T Cell Induction. In: Liston ABT-P@, editor. Progress in Molecular Biology and Translational Science, vol. 136. Waltham, MA, USA: Academic Press (2015). p. 69–97. 10.1016/bs.pmbts.2015.09.001 26615093

[44] Boursalian TE McEarchern JA Law CL Grewal IS. Targeting CD70 for Human Therapeutic Use. In: Grewal IS, editor. Advances in Experimental Medicine and Biology, vol. 647. New York, NY, USA: Springer New York (2009). p. 108–9. 10.1007/978-0-387-89520-8_7 19760069

[45] Jacobs J Deschoolmeester V Zwaenepoel K Rolfo C Silence K Rottey S. CD70: An Emerging Target in Cancer Immunotherapy. Pharmacol Ther (2015) 155:1–10. doi: 10.1016/j.pharmthera.2015.07.007 26213107

[46] Kuka M Munitic I Giardino Torchia ML Ashwell JD. CD70 Is Downregulated by Interaction With CD27. J Immunol (2013) 191:2282–9. doi: 10.4049/jimmunol.1300868 PMC3750068 23913967

[47] Schildknecht A Miesher I Yagita H van den Broek M. Priming of CD8+ Cell Responses by Pathogens Typically Depends on CD70-Mediated Interactions With Dendritic Cells. Eur J Immunol (2007) 37:716–28. doi: 10.1002/eji.200636824 17295392

[48] Ghosh S Köstel Bal S Edwards ESJ Pillay B Jiménez Heredia R Erol Cipe F. Extended Clinical and Immunological Phenotype and Transplant Outcome in CD27 and CD70 Deficiency. Blood (2020) 136:2638–55. doi: 10.1182/blood.2020006738 PMC7735164 32603431

[49] van Montfrans JM Hoepelman AIM Otto S van Gijn M van de Corput L de Weger RA. CD27 Deficiency Is Associated With Combined Immunodeficiency and Persistent Symptomatic EBV Viremia. J Allergy Clin Immunol (2012) 129:787–793.e6. doi: 10.1016/j.jaci.2011.11.013 22197273 PMC3294016

[50] Remedios KA Meyer L Zirak B Pauli ML Truong H-A Boda D. CD27 Promotes CD4 + Effector T Cell Survival in Response to Tissue Self-Antigen. J Immunol (2019) 203:639–46. doi: 10.4049/jimmunol.1900288 PMC6650327 31209102

[51] Peperzak V Veraar EAM Xiao Y Bąbała N Thiadens K Brugmans M. CD8 + T Cells Produce the Chemokine CXCL10 in Response to CD27/CD70 Costimulation To Promote Generation of the CD8 + Effector T Cell Pool. J Immunol (2013) 191:3025–36. doi: 10.4049/jimmunol.1202222 23940275

[52] Ribot JC DeBarros A Pang DJ Neves JF Peperzak V Roberts SJ. CD27 Is a Thymic Determinant of the Balance Between Interferon-γ- and Interleukin 17-Producing γδ T Cell Subsets. Nat Immunol (2009) 10:427–36. doi: 10.1038/ni.1717 PMC4167721 19270712

[53] Dong H Buckner A Prince J Bullock T. Frontline Science: Late CD27 Stimulation Promotes IL-7rα Transcriptional Re-Expression and Memory T Cell Qualities in Effector CD8+ T Cells. J Leukoc Biol (2019) 106:1007–19. doi: 10.1002/JLB.1HI0219-064R PMC6824422 31199542

[54] Erlacher M Strahm B. Missing Cells: Pathophysiology, Diagnosis, and Management of (Pan)Cytopenia in Childhood. Front Pediatr (2015) 3:64. doi: 10.3389/fped.2015.00064 26217651 PMC4500095

[55] Tian C Zhang Y. Purification of Hematopoietic Stem Cells From Bone Marrow. Ann Hematol (2016) 95:543–7. doi: 10.1007/s00277-016-2608-z 26858027

[56] Nolte MA Arens R Van Os R Van Oosterwijk M Hooibrink B Van Lier RAW. Immune Activation Modulates Hematopoiesis Through Interactions Between CD27 and CD70. Nat Immunol (2005) 6:412–8. doi: 10.1038/ni1174 15723067

[57] Ansell SM Flinn I Taylor MH Sikic BI Brody J Nemunaitis J. Safety and Activity of Varlilumab, a Novel and First-in-Class Agonist Anti-CD27 Antibody, for Hematologic Malignancies. Blood Adv (2020) 4:1917–26. doi: 10.1182/bloodadvances.2019001079 PMC7218437 32380537

[58] Reardon D Kaley T Iwamoto F Baehring J Subramaniam D Rawls T. ATIM-23. Anti-CD27 Agonist Antibody Varlilumab in Combination With Nivolumab for Recurrent Glioblatosma (rGBM): Phase 2 Clinical Trial Results. Neuro Oncol (2018) 20:vi6–6. doi: 10.1093/neuonc/noy148.018

[59] A Study of CDX-1127 (Varlilumab) in Patients With Select Solid Tumor Types or Hematologic Cancers (2011). Available at: https://clinicaltrials.gov/ct2/show/NCT01460134 (Accessed: 20th May 2021).

[60] A Dose Escalation and Cohort Expansion Study of Anti-CD27 (Varlilumab) and Anti-PD-1 (Nivolumab) in Advanced Refractory Solid Tumors (2015). Available at: https://clinicaltrials.gov/ct2/show/NCT02335918 (Accessed: 20th May 2021).

[61] Sanborn RE Pishvaian MJ Callahan MK Weise AM Sikic BI Rahma OE. Anti-CD27 Agonist Antibody Varlilumab (Varli) With Nivolumab (Nivo) for Colorectal (CRC) and Ovarian (OVA) Cancer: Phase (Ph) 1/2 Clinical Trial Results. J Clin Oncol (2018) 36:3001. doi: 10.1200/JCO.2018.36.15_suppl.3001

[62] Sanborn RE Pishvaian MJ Kluger HM Callahan MK Weise AM Lutzky J. Clinical Results With Combination of Anti-CD27 Agonist Antibody, Varlilumab, With Anti-PD1 Antibody Nivolumab in Advanced Cancer Patients. J Clin Oncol (2017) 35:3007–7. doi: 10.1200/JCO.2017.35.15_suppl.3007

[63] Villasboas JC Reeder CB Tun HW Bartlett NL Sharon E Laplant B. The DIAL Study (Dual Immunomodulation in Aggressive Lymphoma): A Randomized Phase 2 Study of CDX-1127 (Varlilumab) in Combination With Nivolumab in Patients With Relapsed or Refractory Aggressive B-Cell Lymphomas (NCI 10089/Nct03038672). J Clin Oncol (2019) 37:TPS7570–TPS7570. doi: 10.1200/JCO.2019.37.15_suppl.TPS7570

[64] Lim SH Linton KM Collins GP Dhondt J Caddy J Rossiter L. RIVA - A Phase IIa Study of Rituximab and Varlilumab in Relapsed or Refractory B-Cell Malignancies: Study Protocol for a Randomized Controlled Trial. Trials (2018) 19:619. doi: 10.1186/s13063-018-2996-6 30413184 PMC6230275

[65] Ott PA Pavlick AC Johnson DB Hart LL Infante JR Luke JJ. A Phase 2 Study of Glembatumumab Vedotin, an Antibody-Drug Conjugate Targeting Glycoprotein NMB, in Patients With Advanced Melanoma. Cancer (2019) 125:1113–23. doi: 10.1002/cncr.31892 30690710

[66] Buchan SL Fallatah M Thirdborough SM Taraban VY Rogel A Thomas LJ. Pd-1 Blockade and Cd27 Stimulation Activate Distinct Transcriptional Programs That Synergize for CD8þ T-Cell–Driven Antitumor Immunity. Clin Cancer Res (2018) 24:2383–94. doi: 10.1158/1078-0432.CCR-17-3057 PMC5959006 29514845

[67] Wasiuk A Testa J Weidlick J Sisson C Vitale L Widger J. CD27-Mediated Regulatory T Cell Depletion and Effector T Cell Costimulation Both Contribute to Antitumor Efficacy. J Immunol (2017) 199:4110–23. doi: 10.4049/jimmunol.1700606 PMC5713498 29109120

[68] Vitale LA He LZ Thomas LJ Wasiuk A O’Neill T Widger J. Development of CDX-527: A Bispecific Antibody Combining PD-1 Blockade and CD27 Costimulation for Cancer Immunotherapy. Cancer Immunol Immunother (2020) 69:2125–37. doi: 10.1007/s00262-020-02610-y PMC7511290 32451681

[69] Thiemann M Richards DM Heinonen K Kluge M Marschall V Merz C. A Single-Chain-Based Hexavalent CD27 Agonist Enhances T Cell Activation and Induces Anti-Tumor Immunity. Front Oncol (2018) 8:387. doi: 10.3389/fonc.2018.00387 30298117 PMC6160747

[70] Turaj AH Hussain K Cox KL Rose-Zerilli MJJ Testa J Dahal LN. Antibody Tumor Targeting Is Enhanced by CD27 Agonists Through Myeloid Recruitment. Cancer Cell (2017) 32:777–791.e6. doi: 10.1016/j.ccell.2017.11.001 29198913 PMC5734932

[71] Starzer AM Berghoff AS. New Emerging Targets in Cancer Immunotherapy: CD27 (Tnfrsf7). ESMO Open (2020) 4(Suppl 3):E000629. doi: 10.1136/esmoopen-2019-000629 32152062 PMC7082637

[72] Ramakrishna V Sundarapandiyan K Zhao B Bylesjo M Marsh HC Keler T. Characterization of the Human T Cell Response to In Vitro CD27 Costimulation With Varlilumab. J Immunother Cancer (2015) 3:37. doi: 10.1186/s40425-015-0080-2 26500773 PMC4619281

[73] Ahrends T Babała N Xiao Y Yagita H Van Eenennaam H Borst J. CD27 Agonism Plus PD-1 Blockade Recapitulates CD4+ T-Cell Help in Therapeutic Anticancer Vaccination. Cancer Res (2016) 76:2921–31. doi: 10.1158/0008-5472.CAN-15-3130 27020860

[74] Riccione KA He L-Z Fecci PE Norberg PK Suryadevara CM Swartz A. CD27 Stimulation Unveils the Efficacy of Linked Class I/II Peptide Vaccines in Poorly Immunogenic Tumors by Orchestrating a Coordinated CD4/CD8 T Cell Response. Oncoimmunology (2018) 7:e1502904. doi: 10.1080/2162402X.2018.1502904 30524899 PMC6279317

[75] Auffermann-Gretzinger S Lossos IS Vayntrub TA Leong W Carl Grumet F Blume KG. Rapid Establishment of Dendritic Cell Chimerism in Allogeneic Hematopoietic Cell Transplant Recipients. Blood (2002) 99:1442–8. doi: 10.1182/blood.V99.4.1442 11830498

[76] Nachbaur D Kircher B Eisendle K Lätzer K Haun M Gastl G. Phenotype, Function and Chimaerism of Monocyte-Derived Blood Dendritic Cells After Allogeneic Haematopoietic Stem Cell Transplantation. Br J Haematol (2003) 123:119–26. doi: 10.1046/j.1365-2141.2003.04588.x 14510953

[77] Jardine L Cytlak U Gunawan M Reynolds G Green K Wang X-N. Donor Monocyte–Derived Macrophages Promote Human Acute Graft-*Versus*-Host Disease. J Clin Invest (2020) 130:4574–86. doi: 10.1172/JCI133909 PMC7456218 32453711

[78] Tauro S Craddock C Peggs K Begum G Mahendra P Cook G. Allogeneic Stem-Cell Transplantation Using a Reduced-Intensity Conditioning Regimen has the Capacity to Produce Durable Remissions and Long-Term Disease-Free Survival in Patients With High-Risk Acute Myeloid Leukemia and Myelodysplasia. J Clin Oncol (2005) 23:9387–93. doi: 10.1200/JCO.2005.02.0057 16314618

[79] Shimoni A Nagler A. Optimizing the Conditioning Regimen for Allogeneic Stem-Cell Transplantation in Acute Myeloid Leukemia; Dose Intensity is Still in Need. Best Pract Research: Clin Haematol (2011) 24:369–79. doi: 10.1016/j.beha.2011.05.002 21925090

[80] Saraceni F Beohou E Labopin M Arcese W Bonifazi F Stepensky P. Thiotepa, Busulfan and Fludarabine Compared to Busulfan and Cyclophosphamide as Conditioning Regimen for Allogeneic Stem Cell Transplant From Matched Siblings and Unrelated Donors for Acute Myeloid Leukemia. Am J Hematol (2018) 93:1211–9. doi: 10.1002/ajh.25225 30033639

